# Rat models of spinal cord injury: from pathology to potential therapies

**DOI:** 10.1242/dmm.025833

**Published:** 2016-10-01

**Authors:** Jacob Kjell, Lars Olson

**Affiliations:** 1 Department of Physiological Genomics, Ludwig-Maximilians-Universität München, Munich 80336, Germany; 2 Department of Neuroscience, Karolinska Institutet, Stockholm 171 77, Sweden

**Keywords:** Clinical trials, Rat, Regeneration, Repair, Spinal cord injury

## Abstract

A long-standing goal of spinal cord injury research is to develop effective spinal cord repair strategies for the clinic. Rat models of spinal cord injury provide an important mammalian model in which to evaluate treatment strategies and to understand the pathological basis of spinal cord injuries. These models have facilitated the development of robust tests for assessing the recovery of locomotor and sensory functions. Rat models have also allowed us to understand how neuronal circuitry changes following spinal cord injury and how recovery could be promoted by enhancing spontaneous regenerative mechanisms and by counteracting intrinsic inhibitory factors. Rat studies have also revealed possible routes to rescuing circuitry and cells in the acute stage of injury. Spatiotemporal and functional studies in these models highlight the therapeutic potential of manipulating inflammation, scarring and myelination. In addition, potential replacement therapies for spinal cord injury, including grafts and bridges, stem primarily from rat studies. Here, we discuss advantages and disadvantages of rat experimental spinal cord injury models and summarize knowledge gained from these models. We also discuss how an emerging understanding of different forms of injury, their pathology and degree of recovery has inspired numerous treatment strategies, some of which have led to clinical trials.

## Abstract (precis)

**Summary:** In this Review, we discuss the advantages and disadvantages of the rat for studies of experimental spinal cord injury and summarize the knowledge gained from such studies.

## Introduction

**Fig. 1.**
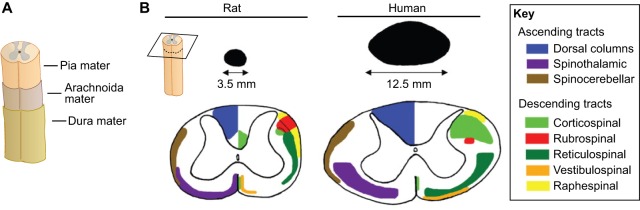
**Spinal cord meninges and tracts.** (A) The spinal cord is surrounded by three meninges: the pia mater, arachnoida mater and dura mater. (B) The rat and human spinal cord differ in terms of size (here cervical cross-section is shown) and the location of the ascending (sensory) and descending (motor) spinal cord tracts. Panel B is reproduced and modified with permission from Watson et al., 2009 (Elsevier).

Spinal cord injury affects millions of people worldwide and typically has life-long consequences (Friedli et al., 2015). In the United States alone, ∼30 individuals sustain a spinal cord injury every day (Gomes-Osman et al., 2016), typically caused by motor vehicle accidents (38%), falls (>22%), violence (13.5%), and sports and recreational accidents (9%). Diseases can also cause or increase the risk of spinal cord injury (National Spinal Cord Injury Statistical Center, 2016). The loss of function that patients experience is dictated by the spinal level of the injury and by the extent and precise anatomical location of damage at this level (Fig. 1). In addition to the immediate consequences caused by loss of motor, sensory and autonomic nervous system functions, secondary processes in the wounded area can aggravate the injury. Later problems include muscle wasting, chronic pain, urinary infections and pressure sores (Abrams and Ganguly, 2015).

Detailed descriptions of the cause and symptoms of spinal cord injury date back to an ancient Egyptian medical text, the Edvin Smith papyrus, from the seventeenth century B.C. (van Middendorp et al., 2010). However, it is only since the latter half of the past century that ways to counteract the effects of spinal cord injury have been subjected to systematic studies in experimental animals (see Box 1). Our evolving understanding of nerve growth inhibition and stimulation, and of the complex immunological, inflammatory and scar-forming reactions that occur in response to CNS injury, have led to the development of several possible pharmacological treatments for these injuries (Silver et al., 2014). These approaches, alone or combined with various cell or tissue transplantation strategies, offer hope that spinal cord injury will become a treatable condition (Olson, 2013, 1997; Tuszynski et al., 2014; Ahuja and Fehlings, 2016; Watzlawick et al., 2016).

**Box 1. Approaches to counteract the effects of spinal cord injury in humans**

• Counteract secondary injury and rescue axons across the site of injury

• Repair spinal circuitry with drugs and with grafts of cells or tissues

• Reactivate surviving, but silent, pathways running across the site of injury

• Use electronic multichannel bridge devices to re-establish connectivity between nerve tracts across the injury

• Rehabilitate patients using state-of-the art equipment for symptom-focused programs

• Use bioimplantable electronic devices coupled to peripheral nerves or the CNS to control muscles

• Use bioimplantable electronic devices coupled to peripheral nerves or the CNS to allow movements of robot-type prostheses

• Use a maneuverable exoskeleton-type device to support locomotion

The rat is a very useful experimental animal for investigating the first five treatment options, and is somewhat less useful for investigating the last three options.

Experimentally, spinal cord injuries in the rat have become the primary model in which to evaluate different experimental treatment strategies (Gomes-Osman et al., 2016; Onifer et al., 2007; Reier et al., 2012). It is from these studies that we have learnt much of what is known about the pathological events that follow spinal cord injury, typically summarized as ‘secondary injury’. Owing to the ease of generating genetic alterations, mice are increasingly used to study the roles of defined proteins in spinal cord injury and repair, and larger mammals are sometimes needed to test that treatments developed in rodents could also work in human-sized species. The rat has nevertheless remained a key experimental animal in spinal cord research. In this Review, we discuss the advantages and disadvantages of the rat for studies of experimental spinal cord injury and summarize the knowledge gained from such studies. Knowledge obtained from rat studies has led to several possible treatment strategies, some of which have led to clinical trials (Table 1).

**Table 1.**
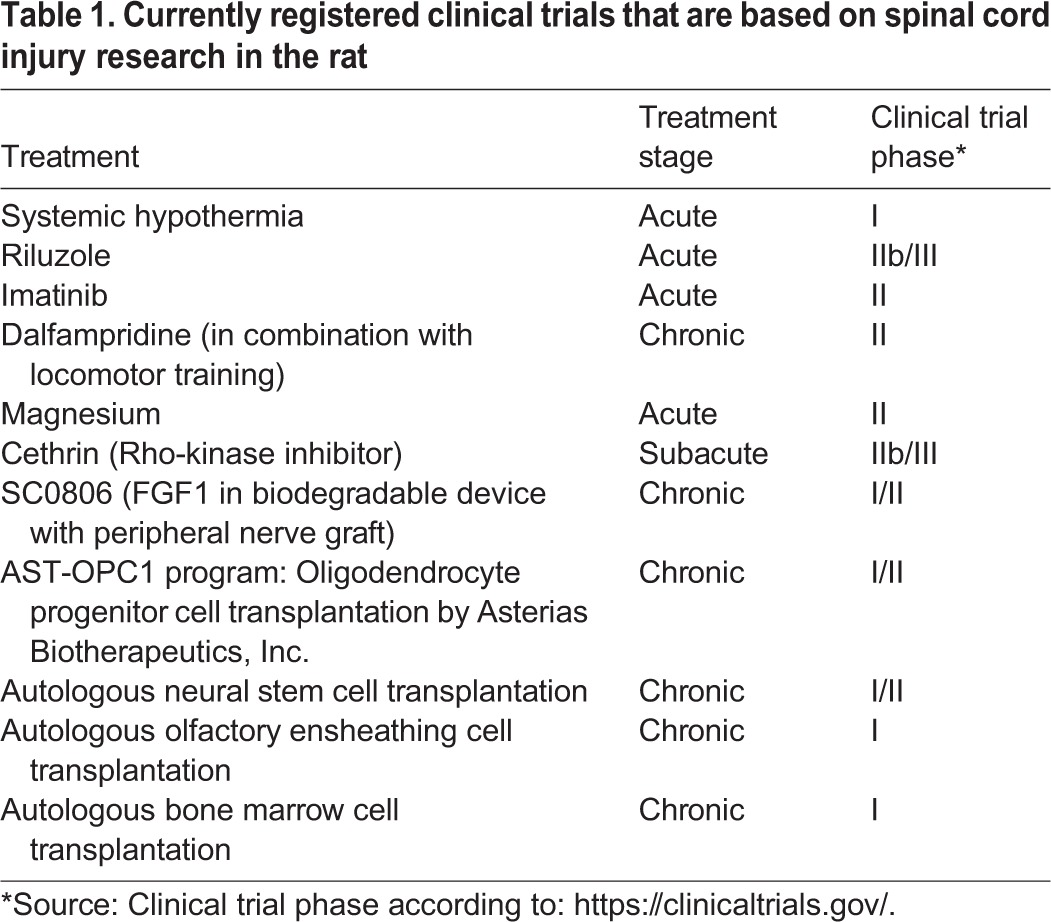
**Currently registered clinical trials that are based on spinal cord injury research in the rat**

## Rat spinal cord injury models the human condition

Animal models of spinal cord injury have historically included cats, dogs and monkeys, prior to the use of rodents. Early animal models generated variable results, and researchers used different behavioral tests to assess the effects of treatment on functional recovery (Silver et al., 2014; Blight, 1983, 1991; Modi et al., 2011; Crowe et al., 1997; Griffiths, 1976; Reier et al., 2012). The first clinical trials of spinal cord injury treatments were based on observations from some of these early models, although disappointing results prompted the field to seek out more standardized (and less costly) rodent models. Importantly, however, the two most commonly used experimental mammals, rats and mice, differ with regard to spinal cord pathology and recovery (Guth et al., 1999; Byrnes et al., 2010). Following injury to the mouse spinal cord, cells proliferate in the injury area, keeping the opposing ends of the transected spinal cord in contact, and typically there is no formation of fluid-filled cysts (Ma et al., 2001; Göritz et al., 2011). There are even reports suggesting that a modest degree of regeneration occurs following a complete transection of the mouse spinal cord (Inman and Steward, 2003). None of these three responses is seen in rats or in humans with injured spinal cords (Metz et al., 2000). Instead, in rats and humans alike, the development of cysts cranial and caudal to the site of injury is common (Bunge et al., 1993, 1997; Josephson et al., 2001). Early formation of fibrotic tissue at the core of the lesion site in rats and humans is typically associated with a breach of the three meninges, allowing fibroblasts to invade the injury site. When spinal cord injury completely severs all axonal connections across the site of injury, motor and sensory function never recover in rats or humans.

The comparisons above demonstrate that rats are preferable to mice for modeling human spinal cord injury. Rats are also robust enough to allow studies not only of the brain, but also of the spinal cord itself, using approaches such as micro-positron emission tomography (microPET), functional magnetic resonance imaging (fMRI) and magnetic resonance spectroscopy (MRS) (Nandoe Tewarie et al., 2010; Fraidakis et al., 1998; Lilja et al., 2006). However, and despite recent progress in the generation of transgenic rats, there is currently almost no alternative to mice for studying the roles of specific genes in CNS injury using transgenic techniques.

## Voluntary walking in rats and humans

It is held by some that intrinsic neural networks in the lumbar spinal cord can maintain non-demanding walking, and that the role of the anatomically prominent human corticospinal tract (CST; see Box 2 for a Glossary of terms, and Fig. 1) is only to turn walking on and off, and to adjust gait as guided by, for example, visual cues. However, in humans, bilateral lesions to the CST have devastating effects on walking (Nathan, 1994), and careful analysis of cortical activity and limb muscle contractions have shown that cortical electrical activity correlates with every step also during undemanding walking in humans (Nielsen, 2003; Petersen et al., 2012). Indeed, detailed comparisons of electroencephalography (EEG; see Box 2) recordings and activity of the leg muscle, musculus tibialis anterior, caused Petersen and coworkers to conclude that “cortical activity does directly contribute to the muscle activity driving uncomplicated treadmill walking” (Petersen et al., 2012). Based on their findings in humans, Petersen et al. emphasized the importance of studying CST rescue and regeneration also in rodent models of spinal cord injury because of the role of the CST in human gait.

**Box 2. Glossary**

**Astrocytic scar:** accumulation of reactive astrocytes around the injury site after a central nervous system (CNS) injury.

**Blood–brain barrier:** a highly selective permeable barrier, formed by the brain's vasculature together with adjacent astroglial perivascular end-feet, that separates circulating blood from the extracellular fluid in the CNS.

**Blood–spinal cord barrier (BSCB):** a highly selective permeable barrier, formed by the spinal cord's vasculature together with adjacent astroglial perivascular end-feet, that separates circulating blood from the extracellular fluid in the spinal cord.

**Cerebrospinal fluid** **(CSF):** the fluid present in the ventricles of the brain, in the central canal of the spinal cord and in the space inside the strong outer meninx (dura mater) that surrounds the CNS.

**Corticospinal tract (CST):** descending motor nerve fibers from the cerebral cortex to the spinal cord that control voluntary movements.

**Craniotomy:** the surgical removal of part of the skull bone in order to expose the brain.

**Cribriform plate:** the bone plate that supports the olfactory bulb and that contains multiple holes through which the olfactory nerves pass.

**Electroencephalography (EEG):** the electrophysiological monitoring of brain activity using electrodes attached to the scalp.

**Fibrotic scar:** the dense and irregular deposition of fibrotic proteins to form scar tissue. These scars share many fibrotic proteins with the basal membrane.

**Proteoglycans:** extracellular matrix protein family that is glycosylated with glycosaminoglycans.

**Rubrospinal tracts:** dorsolateral motor nerve fibers descending from the brain to the spinal cord, which contribute to the control of locomotion and skilled movement. They are considered to be more important for voluntary movement in rodents than in humans.

**Spinal canal:** also called the central canal, the spinal canal contains CSF at the center of the spinal cord and is lined by ependymal cells.

In rats, CST lesions have a less severe effect on gait, but lesions in some of the other pathways descending from the brain, such as the rubrospinal pathway (Box 2) and descending serotonin pathways, are devastating for rat gait (Schucht et al., 2002; Filli et al., 2014). Therefore, even if gait is sustained by partly different pathways in humans and rats, both species depend on pathways that descend from the brain to the spinal cord for their ability to walk. Although gait-like alternating movements of the hind limbs can be elicited in suspended rats, e.g. by a tail pinch (Nygren et al., 1974), following transection of the spinal cord, rats, just like humans with spinal cord injury, will remain permanently paralyzed in their hind limbs (Cheng et al., 1996).

The location of the CST in the rat spinal cord white matter, as well as the location of rat CST axon terminals in gray matter, differs from the CST locations in humans (Watson et al., 2009), which needs to be taken into account when experimental spinal cord lesions are designed. Interestingly, it was recently shown that CST regeneration can be promoted in a rat model of spinal cord injury through the engraftment of spinal cord neural stem cells from either rat embryos or from a differentiated human embryonic stem cell (hESC) line (Kadoya et al., 2016). The authors found that stem cells with a caudal neuronal fate were needed and that the engraftment improved skilled forelimb function.

Even though there is no spontaneous recovery of leg/hindlimb locomotor function after complete interruption of the spinal cord at a low thoracic level in either humans or rats, both species can recover a degree of function after incomplete injury, indicative of structural plasticity in the brain and spinal cord. Such recovery is thought to be mostly due to local structural rearrangements, such as collateral sprouting from remaining axons in gray matter, rather than long-distance regeneration of axons in white matter. This interpretation is supported by recent work, in which recovery from partial spinal cord injury in humans and other primates was compared to that in rats (Friedli et al., 2015). In primates, each descending CST not only contains axons from both the contra- and the ipsilateral cortex but, importantly, the descending axons terminate bilaterally by extensive crossing of branches from one side to the other (decussation) at different levels of the spinal cord (Rosenzweig et al., 2010), whereas such decussations are not found in rats. Hence, after a lateral hemisection of the spinal cord in primates, the remaining motor pathways will not only innervate the non-lesioned side but will also have branches that decussate to innervate the contralateral side of the cord. From these branches, compensatory sprouting can occur, explaining the considerably better recovery from lateralized spinal cord injury in primates compared to rats (Friedli et al., 2015). However, similar compensatory sprouting can also be studied in rats, using partial lesions of the CST (Courtine et al., 2008; Raineteau and Schwab, 2001). Indeed, and probably due to the smaller size of the rat CNS compared to that of humans, loss of innervation of one side of the spinal cord in rats can be compensated for by sprouting of fibers from the intact side; these fibers cross the midline at different levels of the descending systems in response to the injury (Raineteau and Schwab, 2001; Filli et al., 2014).

## Dormant residual pathways: a hope and a pitfall

Remarkable recent work by Harkema and coworkers highlights the importance of studying repair strategies in rats with anatomically truly complete disconnection between cranial and caudal parts of the spinal cord at the site of injury, if the purpose is to prove that reconnection therapies are possible. This is because even a minute number of remaining nerve fibers across the site of injury might contribute to recovery. Harkema and coworkers studied adult humans with complete motor paraplegia. They implanted multi-electrode stimulator plates on the dura (Fig. 1A), over the lumbar spinal cord of each patient, and found that, with the right pattern of stimulation, four of four patients recovered a degree of motor control of their legs (Angeli et al., 2014). Although movements were modest in the beginning, mentally ‘finding’ lost pathways allowed patients to train and markedly improve their leg motor functions. In one case, training allowed a patient to have a degree of leg control even when the stimulator was turned off. It was found that the patients could carry out leg movements in response to visual or auditory commands, providing proof that the movement commands came from the brain. The findings suggest that, after spinal cord injury, there might be remnants of descending axon pathways that cannot be used owing to loss of myelin or because the number of axons is too low. Epidural stimulation of the lumbar cord might increase the sensitivity of the local spinal cord circuitry such that very weak descending signals begin to produce effects. Once this happens, the circuitry can become strengthened by voluntary training, which increases connectivity through structural synaptic plasticity and which might also induce re-myelination.

Electrical stimulation has been shown to promote motor recovery in both humans and rats. The fact that electrical stimulation (neuromodulation) can be a therapy for both incomplete and functionally complete injuries in humans (Carhart et al., 2004; Field-Fote, 2002) demonstrates the need to study neuromodulation after spinal cord injury in rats to further understand the physiological principles that underlie improved motor function (Wenger et al., 2014, 2016). This treatment modality is particularly exciting given the possibilities of combining it with tissue sparing and/or regenerative therapies.

It is obvious from the work of Harkema and coworkers that, in order to prove that a repair strategy, in the sense of bridging a physical gap, has been successful, the experimenter must prove that the spinal cord was completely transected prior to the repair procedure. The rat is a better experimental animal than the mouse when it comes to testing such repair strategies, owing to the ‘risk’ of spontaneous recovery in mice. One convincing way to demonstrate complete disconnection in a repair model is to remove a thin segment of the spinal cord. Transverse histological sections of this segment can then demonstrate spinal cord gray and white matter, completely surrounded by dura, proving completeness of the injury (as demonstrated e.g. in Cheng et al., 1996).

## Different types of experimental spinal cord injury

Most types of spinal cord injuries seen in humans can be replicated in adult rats. These include complete and incomplete spinal cord injuries at different levels. However, at the cervical level, ethical and medical arguments prevent bilateral injuries from being modeled in rats because these injuries would paralyze both forelimbs and hindlimbs. However, defined unilateral injuries at the cervical level can be studied. At low thoracic levels, complete spinal cord injury causes permanent paralysis of the hindlimbs and a corresponding impairment of sensory and autonomic system functions. Animals with low-level thoracic injuries ambulate by using their forelimbs. Provided that assisted bladder emptying is used as long as needed, and that other forms of care such as treatment of urinary infections and of sores is also provided, these animals can be studied for several months.

Lesions can also be created in the lumbar, or even the sacral, spinal cord of rats. Sacral injuries tend to cause symptoms that are restricted to the tail but, because tail positions and types of tail movements are characteristically coupled to different forms of locomotion in rats, sacral lesions can also be used to monitor recovery of function following treatments (Bennett et al., 1999).

**Fig. 2.**
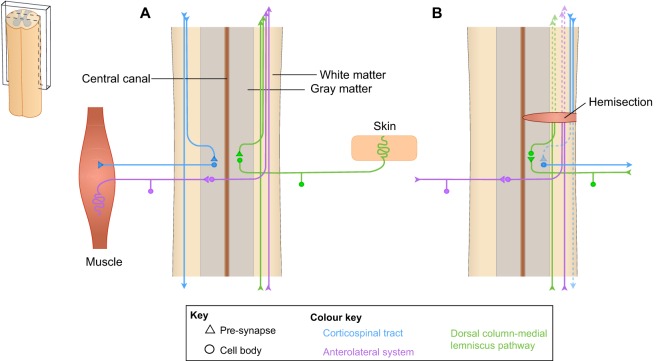
**Spinal cord circuitries and loss of innervation following injury.** Schematic drawings of longitudinal sections of spinal cord (left), demonstrating (A) selected descending (illustrated on the left, in blue) and ascending (illustrated on the right, in green and purple) circuitry of the uninjured spinal cord, and (B) injury to the spinal cord, which causes loss of motor and sensory function depending on the location and severity of the injury. The loss of long fiber tracts (dashed lines) caused by hemisection of the spinal cord is shown.

Precision lesions can be generated using knives or scissors, and can model certain forms of human injury, such as knife attacks (see Fig. 2B). However, spinal cord injuries in humans are typically caused by falls or other forms of physical impact that crush the bony canal and compress the spinal cord. This is typically modeled in rats by first removing part of the bony wall of the spinal canal (Box 2) and then subjecting the exposed spinal cord to compression or contusion injuries (Rosenzweig and McDonald, 2004).

Compression injuries are typically done with a clip, but forceps and balloon injuries (where a tiny balloon is inserted into the spinal canal and expanded to cause pressure on the spinal cord) have also been used (Rivlin and Tator, 1978; Vanický et al., 2001; Blight, 1991). Prolonged compression has been shown to aggravate outcome. In line with such observations, it has been shown in rats that decompression reduces secondary pathology and improves recovery (Carlson et al., 1997; Dimar et al., 1999; Dolan et al., 1980). Studies in patients mainly support a neuro-protective effect of decompression measures (Fehlings and Perrin, 2005). Arguably, decompression therefore constitutes the only current ‘treatment’ in humans, and is only applicable to certain forms of spinal cord injury.

Contusion injuries have typically been modeled using a weight-drop device (Gruner, 1992), with current versions adjusting and recording the force produced by the piston (Scheff et al., 2003). These contusion injuries, caused by an accelerating rod impacting the spinal cord, are considered to rather faithfully model human impact injuries to the spinal cord (Metz et al., 2000). Experimentally, a gradual increase in force causes a reproducibly increased graded loss of tissue and motor function in rats.

Notably, rats recover from spinal cord injury in a strain-specific manner (Mills et al., 2001), and even substrain-specific differences in functional outcome and tissue sparing exists (Kjell et al., 2013). The reasons for these differences are not well understood but seem to be genetic. Moreover, the anatomical location of the spinal fiber tracts differs between humans and rats (Fig. 1) (Watson et al., 2009). Indeed, there is evidence that axonal tracts might also differ somewhat in their location between rat substrains (Clark and Proudfit, 1992). Thus, even substrain selection becomes important for the interpretation of results in experimental spinal cord injury models.

## Lost functions and how to measure them

Rats develop symptoms similar to those seen in humans after spinal cord injury, and there are several behavioral tests available to assess the loss and recovery of sensory and locomotor functions. Although humans with spinal cord injury typically rate the recovery of bladder, bowel and sexual functions higher than the recovery of gait, the single most visible sign of recovery from spinal cord injury, in humans and rats alike, is recovery of the ability to walk, indeed a very important function to recover.

The Basso, Bresnahan and Beattie (BBB) score of hindlimb motility during walking has become a universal measure of functionality following the induction and treatment of spinal cord injury in rats (Basso et al., 1996). It turns out that the scores from 0 to 21 (spanning from complete flaccid paraplegia to normal function) behave almost as linear, normally distributed data (Scheff et al., 2002), and sensitively detect levels of functionality. Trained personnel score injuries very similarly, demonstrating this method's reliability, in addition to its validity; this scoring method has been cited in perhaps a thousand published studies to date. The BBB sub-score (Lankhorst et al., 1999) focuses on additional functional deficits (such as toe clearance, paw positioning, instability and tail position), and is particularly helpful when the injury is moderate to severe. In addition to walking, swimming and wading can also be analyzed by other scoring systems in rats (Xu et al., 2015; Zörner et al., 2010).

The battery of behavioral tests in rat studies of spinal cord injury focus on motor abilities and also include grid walk tests, balance tests and many additional tests (Onifer et al., 2007). Gait parameters can be observed and analyzed from video recordings from below as animals walk on a glass floor. Regular walking with three feet always touching the floor can be carried out in six different ways, and all can be observed in rats (Cheng et al., 1997). In addition to scoring by trained observers, there are automated gait analysis methods for rats (and mice), including the Catwalk (Hamers et al., 2001), which provides a number of different gait parameters derived from data analysis of patterns and timing of foot positions and foot prints. Other sophisticated ways to record and analyze leg movements from video recordings use identifiable markers on leg joints (van den Brand et al., 2012), as well as electromyography. Partially paraplegic rats can also be trained to perform bipedal walking, by letting the rat hang in a body support device that allows the hindlimb feet to touch the ground and perform walking movements that can be scored (Fraidakis et al., 2004) or analyzed from video recordings using dedicated software (van den Brand et al., 2012).

Sensory and autonomic functions are also important to monitor, using tools such as the von Frey test (Chaplan et al., 1994) and tests for sensitivity to heat and cold (Erschbamer et al., 2007), as well as the hindlimb plantar placing reflex, thought to be an indicator of involvement of the CST (Donatelle, 1977). The head scratch test is a way to test descending (but not ascending) activity that travels across the site of injury as it elicits hindlimb and tail movements (Fraidakis et al., 2004). Sensory disturbances such as allodynia (a condition in which touch elicits pain) and neurogenic pain (a condition featuring pathological increases in pain) can manifest in patients. Mild spinal cord injuries in rats typically cause a longer period of hypersensitivity to mechanical and thermal stimuli (although some rats display loss of sensory function) (Kjell et al., 2013). Like human patients, rats with severe spinal cord injuries can develop allodynia (Bruce et al., 2002; Hofstetter et al., 2005).

## To rescue what can be rescued

Experimental contusion spinal cord injuries in rats have been extensively used to understand the complex secondary events that follow the primary injury and that typically aggravate outcome. These studies are important because the secondary injury phase is one that can be targeted pharmacologically to decrease permanent damage after spinal cord injury.

The question of whether progressive neuronal cell death occurs after spinal cord injury was resolved by studies in the rat, which showed that apoptosis (programmed cell death) is a major cause of post-injury neuronal death, whereas other neurons undergo necrosis (Crowe et al., 1997; Lou et al., 1998; Emery et al., 1998; Zhang et al., 1997; Tator, 1995; Beattie et al., 2000). Neural apoptosis is prevalent 3-8 h after injury, whereas, after 24 h, such neuronal loss no longer occurs (Liu et al., 1997; Schumacher et al., 2000; Citron et al., 2000). Apoptosis also affects glial cells and inflammatory cells, and extends beyond the 24 h window reported for neurons. Apoptosis of all these cell types is also seen after human spinal cord injury, although less is known about the time course during which the different cell types undergo apoptosis in the injured human spinal cord (Emery et al., 1998).

The fact that there seems to be a time window during which both neurons and glial cells can be saved from programmed cell death offers an opportunity for protective therapeutic intervention. It has also been postulated that cytotoxic effects caused by the release and leakage of excitatory signal substances and other potentially toxic molecules released by damaged cells contribute to cell death soon after injury (Faden and Simon, 1988). In line with this, it has been shown that Na^+^- and NMDA-channel antagonists improve recovery from spinal cord injury in rats (Faden et al., 1990; Teng and Wrathall, 1997). Riluzole – a treatment for amyotrophic lateral sclerosis – which blocks Na^+^ channels and inhibits glutamate release, also improves recovery from spinal cord injury in rats (Schwartz and Fehlings, 2001) and is currently in a Phase 2b/3 clinical trial for spinal cord injury (Fehlings et al., 2015). The advancement of Riluzone into clinical trials for spinal cord injury was based on studies in rats, although its neuroprotective function has also been confirmed in other species and models (Nagoshi et al., 2015).

**Fig. 3.**
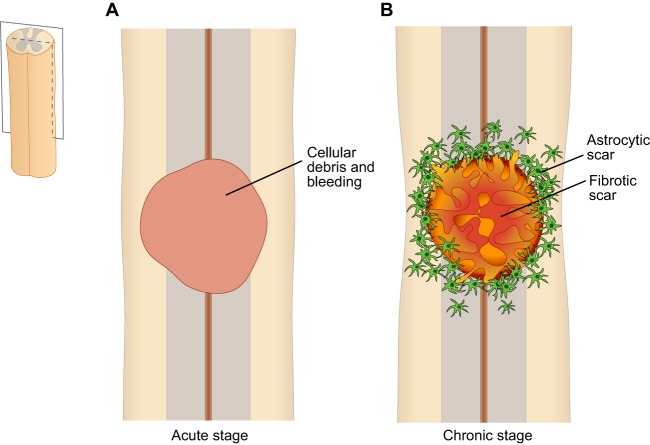
**Stages after spinal cord injury.** Schematic drawings of longitudinal sections of the rat spinal cord after injury. (A) In the acute stage (e.g. 1 day after injury), the lesioned area is filled with debris from dead cells and fills with fluid caused by bleeding. (B) In the chronic stage (e.g. 6 weeks after injury), the lesioned area becomes filled with a fibrotic scar and is surrounded by a dense rim of reactive astrocytes in the spared (not directly injured) white and gray matter. The fibrotic core becomes denser with time and contains the cells that deposit the extracellular matrix, as well as inflammatory cells (mostly macrophages) (not shown).

CNS injury, not least spinal cord injury, will also lead to a breach of the blood–brain barrier (see Box 2), and also to bleeding and edema, all of which impair blood circulation and cause ischemia because of damaged blood vessels and increased pressure inside the dura mater (Phang et al., 2015) (Fig. 3A). In stroke, further neurological impairment can take place if thrombolytic treatment with recombinant tissue plasminogen activator (tPA) is administered beyond a narrow time window, owing to increased edema and vascular permeability. Investigations into the mechanism that underlies this unwanted side effect of tPA have revealed that tPA causes proteolytic activation of latent platelet-derived growth factor-CC (PDGF-CC), and that the loss of blood–brain-barrier integrity is a consequence of PDGF-CC activating PDGFR-α on astrocytic end-feet lining the capillary walls (Su et al., 2008, 2009; Fredriksson et al., 2015).

**Fig. 4.**
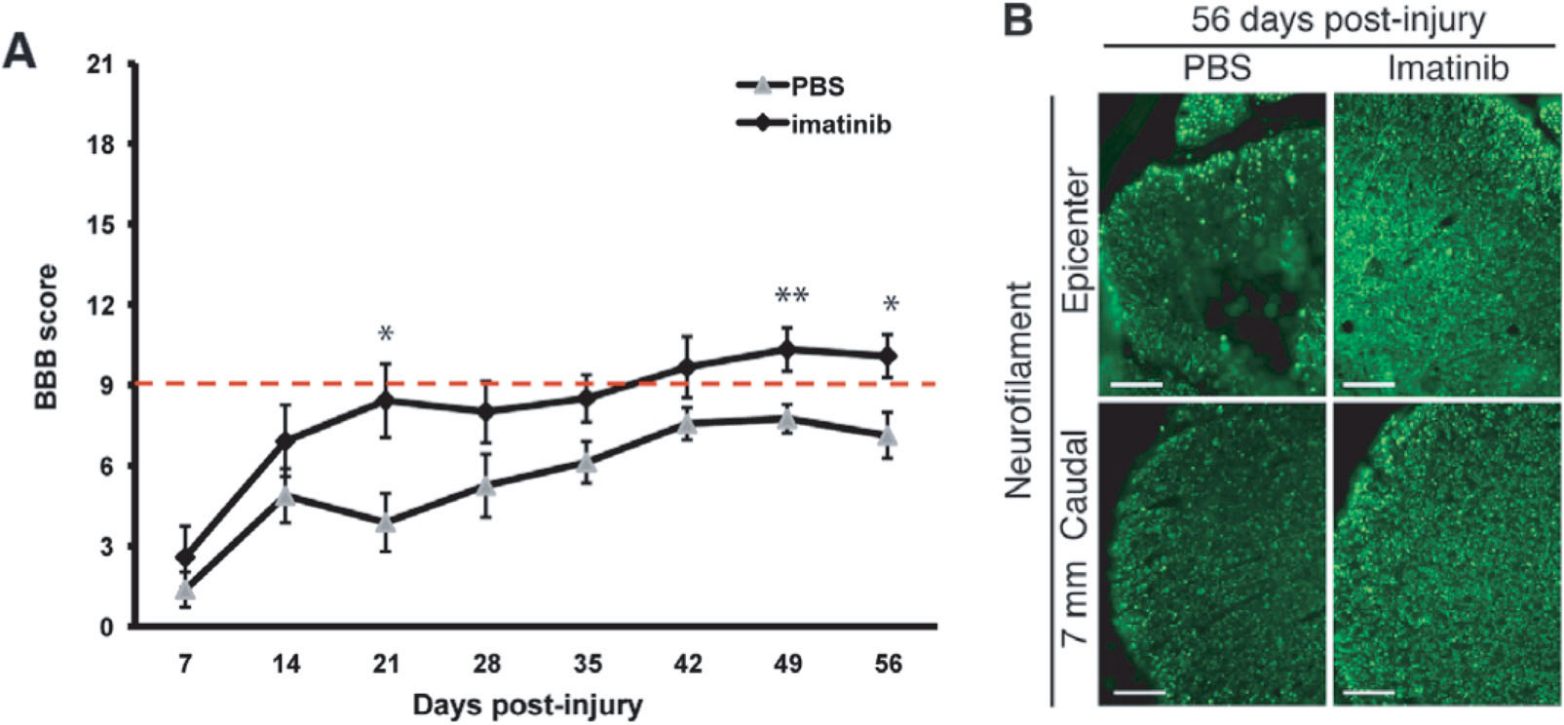
**Imatinib treatment is protective in rats.** We (Abrams et al., 2012) examined the effects of oral imatinib treatment in rats after a spinal cord contusion injury. The Basso, Bresnahan and Beattie (BBB) scoring method (see main text) was applied to measure hindlimb locomotor function. Treatment with phosphate-buffered saline (PBS) was used as a control. (A) BBB scores demonstrate an improvement in hindlimb locomotion with 5 days of oral imatinib treatment initiated 30 min after injury. Rats with a locomotor score above the dashed red line (a BBB score of 9) can support their own weight on their hindlimbs, whereas those below cannot. *P<0.05 and **P<0.01. (B) Micrographs illustrating axon (neurofilament) density in sections of the spinal cord from animals that received PBS or imatinib treatment. The pan-neurofilament marker SMI-312 (green) defines the magnitude of neurofilament (and hence axon) sparing at the injury site and caudal to the injury site 8 weeks after spinal cord contusion injury. Treatment with imatinib (right-hand boxes) rescues many neurofilament-positive axon profiles that are lost in the untreated injured spinal cords 8 weeks after injury. Scale bars: 100 μm. Reproduced with permission from Abrams et al. (2012).

The studies by Su et al. in a stroke model (Su et al., 2008) have prompted investigations into the therapeutic potential of imatinib, a PDGFR-α antagonist and cancer drug, in acute spinal cord injury (Kjell and Olson, 2015). A first proof-of-concept study for imatinib involved rats with weight-drop injury to the lower thoracic spinal cord (Abrams et al., 2012). These rats were treated with the drug as soon as they fully awakened (≈30 min) following anesthesia for spinal cord injury surgery. Treatment continued during the period of extensive blood–spinal-cord barrier (BSCB; see Box 2) permeability after injury [the spatiotemporal aspects of BSCB permeability is well defined in rats following spinal cord injury (Figley et al., 2014; Popovich et al., 1996; Cohen et al., 2009)]. Both functional (locomotor function and bladder function) and histological parameters (tissue sparing, axonal sparing, astrogliosis, inflammation and BSCB permeability) were improved by treatment with imatinib following spinal cord weight-drop injury (Abrams et al., 2012) (Fig. 4). Cyst formation was also attenuated. Because imatinib is in clinical use as a cancer treatment, there is ample data on its dose-response toxicity in rats compared to humans (European Medical Agency, 2004), which allows feasible and translationally relevant doses to be estimated for rats. The dosage used in the rat is estimated to correspond to 800 mg of imatinib once per day in humans. In humans, both 400 and 800 mg are considered effective in the treatment of chronic myeloid leukemia, and these dosages have similar side-effect profiles.

In a second set of experiments of imatinib as a treatment for acute spinal cord injury in rats, our group studied how long after injury it was possible to wait before starting imatinib treatment and still obtain positive results. We also studied recovery of sensory function (mechanical and thermal), inflammatory activity in lymphoid organs versus at the injury site, and potential biomarkers in serum (Kjell et al., 2015). Using rats allows repeated blood sampling for multiple biochemical analyses of, for example, cytokines, growth factors or cell-specific proteins, with only a few days between sampling. This makes it possible to monitor time courses of potential biomarkers of effective blood levels of candidate drugs in individual animals. Using this approach, we identified three surrogate markers of imatinib bioactivity. Importantly, alterations in blood cytokine levels following imatinib treatment (Kjell et al., 2015, 2016) correlated well with results from cancer patients on chronic imatinib treatment (Hayashi et al., 2012), providing further support for the rat as a relevant animal model also when it comes to assessing drug-elicited effects on inflammatory processes. With respect to the acute release of cytokines in the cerebrospinal fluid, rats and humans respond similarly; however, the cytokine release profile in humans is extended over time relative to that in the rat (Kwon et al., 2010). To date, the cancer drug imatinib has been shown to be protective in several rodent studies of CNS injuries and disorders (Adzemovic et al., 2013; Merali et al., 2015; Su et al., 2015; Ma et al., 2011; Abrams et al., 2012), and a first report from a Phase 2 clinical trial with imatinib after stroke suggests that imatinib might be clinically effective (http://www.medscape.com/viewarticle/863881).

## Inflammation: a regulator of degeneration and regeneration

Another hallmark of secondary spinal injury is the inflammatory response. This response consists of an almost immediate response mediated by resident microglia and the subsequent infiltration of different populations of immune cells. In the rat, neutrophils are the first to infiltrate the spinal cord after injury, with the number of cells increasing between 3 and 6 h after injury (Taoka et al., 1997). This time window suggests that neutrophils might have a role in the apoptosis of neurons. In support of this, reducing neutrophil infiltration with anti-CD11d antibodies improves recovery after spinal cord injury in rats and mice (Geremia et al., 2012; Bao et al., 2004), although neutrophil depletion has been found to impair recovery (Stirling et al., 2009). At 3 days after injury, when blood-borne monocytes start to infiltrate the cord in rats, neutrophils are no longer present (presumably owing to apoptosis) (Fleming et al., 2006).

Macrophages become chronically present in the injured spinal cord of rats and humans. Rat studies have determined that blood-monocyte-derived macrophages have two peaks, at around 7 and 60 days after injury, which suggests temporally separated waves of infiltration (Popovich et al., 1997; Blight, 1992; Beck et al., 2010). Furthermore, macrophage responses, including those of microglia, are heterogeneous. One group of macrophages seems to mainly promote degeneration (M1), whereas another seems to mainly promote regeneration (M2) (Kigerl et al., 2009), although there seems to be a continuum of cell types, rather than distinct classes. Other macrophage subclasses have been proposed to have specific functional properties (Heppner et al., 2015). Indeed, transplanting macrophages with an M2 phenotype or manipulating endogenous macrophages towards an M2 phenotype attenuates pathology, promotes regeneration and improves functional recovery after spinal cord injury in rats (Hawthorne and Popovich, 2011; Guerrero et al., 2012; Rapalino et al., 1998; Nakajima et al., 2012; Miron et al., 2013).

Targeting myelin basic protein (MBP) with autoantibodies and/or MBP-competent T cells has been found to improve outcome in experimental spinal cord injury in rats (Huang et al., 1999; Hauben et al., 2000a,b; Jones, 2004). In general, much less is known about both T- and B-cell responses to spinal cord injury and about their impact on injury compared to other immune cells, such as macrophages. T cells progressively infiltrate injured spinal cords starting at 12 h after injury and peaking at day 7 in rats (Popovich et al., 1997). Current evidence suggests that these cells form part of the chronically resident population of inflammatory cells after injury (Beck et al., 2010). Autoantibodies have been found in serum after spinal cord injury in humans, indirectly pointing to the presence of B cells (Hayes et al., 2002).

Infections are associated with a worsened outcome after spinal cord injury in rats and humans (Failli et al., 2012). However, certain asymptomatic infections might also improve functional recovery in rats (Kjell et al., 2016); it is thus necessary to know the health status of rats used in experimental studies. To what extent asymptomatic infections in humans contribute to the variability of recovery from spinal cord injury is not known.

## Progressive scarring

Axonal regeneration across the site of any focal experimental spinal cord injury is limited by the build-up of physical barriers, as well as by molecular inhibition of nerve growth and by an insufficient presence of nerve growth stimulation factors (Silver et al., 2014). In addition, neurons with long axons, such as the CST neurons, can obtain sufficient neurotrophic support from axon branches that innervate areas proximal to the site of injury, and might therefore not ‘need’ to regenerate the part of the axonal arborization lost in a spinal cord injury to promote survival. In fact, studies show that CST neurons survive long-term after spinal axotomy (McBride et al., 1990). The process of incomplete wound repair of the spinal cord involves scarring, as in most other organs and tissues of mammals. In the spinal cord, however, scarring consists of two components: the glial (astrocytic) scar and the fibrotic scar (Box 2; Fig. 3B).

The main components of the glial scar are hypertrophic reactive astrocytes around the lesion, which progressively form an astrocytic ‘scar’. The astrocytic scar constitutes a physical barrier and expresses molecules that are inhibitory to axon growth; nonetheless, a limited number of axons may pass this barrier (Anderson et al., 2016; Cregg et al., 2014). Reactive astrocytes are typically characterized by increased amounts of the intermediate filament GFAP (glial fibrillar acidic protein), and histochemical comparisons between rodents and humans are based on this marker. In rats, reactive astrocytes cluster at the border of the lesion by 1-2 weeks after injury; after 2-3 weeks, the astrocytic ‘scar’ has matured. Observations from human spinal cord injuries have found such scar formation to be a late occurrence (4-6 months after injury), although astrocyte reactivity can be found much earlier (1-2 weeks after injury) (Norenberg et al., 2004; Buss et al., 2007). This type of ‘scar’ is considered to be chronic, and it has been reported to be present in humans 30 years after spinal cord injury (Buss et al., 2004). In mice, there is proliferation of juxtavascular astrocytes and some astrocytes differentiated from ependymal cells of the central canal, which make up the dense astrocytic scar (Bardehle et al., 2013; Barnabé-Heider et al., 2010). Early removal of the astrocytic scar, however, has been found to increase the size of the lesion area and to reduce functional recovery in mice (Faulkner and Keirstead, 2005; Anderson et al., 2016). Reactive astrocytes might thus have both positive and negative effects on regeneration, and perhaps different properties depending on the time after injury or their interaction with other cells. Astrocytes produce proteoglycans (Box 2) under physiological conditions and increase the production of some proteoglycans following injury (Anderson et al., 2016). However, other cells that are present at the lesion also contribute substantially to the production of proteoglycans.

The mature astrocyte scar around the injury site is to some extent associated with chondroitin sulfate proteoglycan (CSPG) deposition in both humans and rats (Buss et al., 2007, 2009), and CSPG reduces axon growth *in vitro* (McKeon et al., 1991). Such effects might be both physical and ligand-receptor specific because CSPG binds to Nogo receptors 1 and 3 (Dickendesher et al., 2012), which are present, for example, in CST neurons (Karlsson et al., 2013). To test the hypothesis that the enzymatic removal of CSPG might benefit axonal growth after spinal cord injury, Bradbury et al. treated rats that had a spinal cord injury with chondroitinase ABC and reported improved functional recovery and increased axon regeneration across lesion sites following treatment (Bradbury et al., 2002). It should be noted that there seems to be some differences between humans and rodents with respect to the location and timing of glycosaminoglycan deposition (from e.g. CSPGs) and also the identity of the proteoglycans associated with the glycosaminoglycans, although investigations of the human spinal cord are currently limited to histochemical observations across a few time points (Bruce et al., 2000; Buss et al., 2009).

The fibrotic scar is typically extensive after most spinal cord injuries. This scar component has been associated with a breach of the meninges and with fibroblasts, which produce a dense extracellular matrix (ECM) (Hermanns et al., 2001). However, contusion injuries, which occur without an overt breach of the meninges, also result in the progressive formation of a fibrotic scar in rats and humans (Loy et al., 2002; Silver and Miller, 2004). Thus, axonal regeneration becomes physically inhibited by the arrival of different cell types to the injured area and by the dense deposition of ECM components. In rats, the fibrotic scar consists of ECM proteins found in the basement membrane, including collagen 4, laminin and fibronectin (Loy et al., 2002; Stichel et al., 1999). Prior to the maturation of the fibrotic scar, at 3-7 days after injury, angiogenesis occurs at the core of the lesion. Although basement membrane sheaths are formed, many do not associate with endothelial cells, and revascularization remains poor (Loy et al., 2002). Although many nerve fibers associate with laminin sheaths at 2 weeks after injury, such fibers are later found retracted. Recent studies in mouse implicate pericytic cells as contributing to scar tissue, suggesting a role for angiogenesis in fibrotic scar formation (Göritz et al., 2011; Soderblom et al., 2013). In rats, fibrotic-scar-forming cells have been described as a type of fibroblast or fibrocyte (Sroga et al., 2003); however, these scar-forming cells have never been properly defined owing to the lack of genetic models. Whether these two cell types are corresponding cell populations remains unknown.

## Repair strategies developed in rats

The striking difference between lack of axon regeneration in the CNS and its presence in the peripheral nervous system (PNS) of adult mammals seems not to be a principal difference between CNS and PNS neurons, but rather a difference of the their respective environments. Thus, whereas Schwann cells, which are present in the PNS, support regeneration in many ways, oligodendroglial cells, which are present in the CNS, inhibit regeneration. Indeed, as discussed below, many types of CNS neurons will readily regenerate axons when provided with a Schwann cell environment. The effective inhibitory mechanisms of the white matter of the CNS also seem difficult to overcome through the delivery of neurotrophic factors (Schwab, 2010), although such treatment might have nerve growth stimulatory effects in gray matter circuitry.

A few repair strategies tested in rats have resulted in the return of a degree of function after complete spinal cord transection. Importantly, this return of function has been shown to be lost again when a new transection of the spinal cord is made at the same or at a proximal level of the spinal cord. One such repair protocol is based on multiple bridges across the injury, formed by the autologous engraftment of pieces of peripheral nerve (Cheng et al., 1996; Fraidakis et al., 2004; Lee et al., 2010, 2013; Depaul et al., 2015). An alternative to the use of a growth-promoting Schwann-cell rich conduit, such as a piece of peripheral nerve, is to establish a relay by implanting neural stem cells that become neurons and extend axons both in cranial and caudal directions (Lu et al., 2012; Kadoya et al., 2016). Although a relay can help recover limb movement to an impressive extent, it might in itself be limited in its functional potential for finer motor skills. However, such transplants might also act as growth substrate for the regeneration of descending and ascending axons, eventually allowing the recovery of motor skill and sensory functions (Lu et al., 2012; Kadoya et al., 2016). Other cell-grafting strategies involve using Schwann cells (Guest et al., 1997; Williams et al., 2015; Bunge, 2016), olfactory ensheathing cells (Tabakow et al., 2013; Raisman and Li, 2007), other stem cells, embryonic CNS cells (or tissues), cells from the immune system (Rapalino et al., 1998) and cells transfected to release neurotrophic factors (Lu et al., 2003). In principle, these repair strategies should also be applicable to chronic spinal cord injury in humans, because a disconnected spinal cord distal to a lesion will remain viable for decades after injury in humans (Bunge et al., 1961).

## From experiments in rats to human trials

Neurogenesis occurs in the adult mammalian olfactory epithelium in rodents and primates, including humans (Hahn et al., 2005; Borgmann-Winter et al., 2009). Axons can grow from their nerve cell bodies of origin in the olfactory mucosa all the way to the olfactory bulb (Graziadei and Graziadei, 1979; Harding et al., 1977; Monti Graziadei et al., 1980), during the course of which the axons cross the interface between a PNS and a CNS environment. In fact, if the olfactory nerve is injured, it is not the cut axons that regenerate. Instead, axons from newly formed neurons in the olfactory mucosa grow all the way from the olfactory epithelium, through the cribriform plate (see Box 2) to the olfactory bulb to engage in forming glomeruli (Schwob, 2002). This remarkable ability of adult olfactory nerve axons to grow and extend to the olfactory bulb has been ascribed to the presence of a specific population of glial cells that have particular axon growth and guidance properties: the olfactory ensheathing cells (Doucette, 1990, 1995; Ramón-Cueto and Valverde, 1995). Studies in rats suggest that olfactory ensheathing cells can be used to enable regeneration and thus the repair of CNS injuries, including spinal cord injury (Li et al., 1997). Recently, numerous experimental studies have confirmed the axon growth-promoting effects of olfactory ensheathing cells, giving rise to the idea that these cells could be obtained from the olfactory mucosa and proliferated for use in individuals with spinal cord injury (Jani and Raisman, 2004). In parallel, the use of peripheral nerve grafts as Schwann-cell-containing conduits that promote the regeneration of CNS axons, as first observed by Cajal's student Tello (Tello, 1911) and used as a way to demonstrate long-distance regeneration of spinal cord axons (Richardson et al., 1980), provided the first evidence of partial functional recovery from a complete spinal cord injury in an adult mammal (rat) (Cheng et al., 1996). Thus, engraftment strategies based on olfactory ensheathing cells and/or Schwann cells/peripheral nerves are of clinical interest.

The engraftment of autologous olfactory ensheathing cells cultured from the human olfactory epithelium has been tested as a treatment for chronic spinal cord injury (Tabakow et al., 2013). A recent case report suggests marked improvements in an individual with a knife injury to the spinal cord, who was treated with a combination of peripheral nerve grafts (from the sural nerve) and engraftments of olfactory ensheathing cells (Tabakow et al., 2014). In this patient, olfactory epithelium biopsies could not be used as a source of olfactory ensheathing cells owing to chronic infection. Instead, one olfactory bulb was removed via a frontolateral craniotomy (Box 2), and olfactory ensheathing cells were cultivated from this tissue, which is known to be a better source of olfactory ensheathing cells, as well as of other olfactory glial cells, than the olfactory mucosa. This single case, in which two repair strategies, both developed in rats, were combined, resulted in remarkable functional recovery. Although the recovery is well documented, the relative roles of the various procedures have not been determined and questions have been raised as to the mechanisms behind the recovery in this case report, particularly the role of long-distance axon regeneration (Guest and Dietrich, 2015). Additional patients are needed to document the possible benefits of this combined approach.

## Limitations and future possibilities

Despite the many advantages as a spinal cord injury model with translational value, the rat model remains far from perfect. Genetic modification is more difficult in the rat than in the mouse and, even though new methods have recently resulted in some commercially available genetically modified rats, mice remain the animal of choice for studies involving genetic manipulation. Although rats are larger than mice, the rat is still a very small animal compared to humans. Hence, long-distance axon regeneration, as needed in humans to repair spinal injuries, cannot be directly studied in the rat. Indeed, experimental results from rodent studies that report improved axonal growth (e.g. because of axons bridging the lesion site) might misinform us, because the volumes of gray matter that need reinnervation are much larger in humans than in rats. Human recovery after spinal cord injury is also slower than in the rat. Spontaneous recovery in humans is not considered to reach a plateau until 6-12 months after injury. The recovery of rats, on the other hand, typically plateaus ∼6-8 weeks after injury. The different time scales might reflect the longer regeneration distances needed in humans, compared to rats. As much as the short recovery period in rats is an advantage with respect to advancing experimental research, this difference in recovery periods might have implications for the investigation of therapies, particularly for treatments that need to be implemented during a specific time window. Data concerning secondary injury in humans also point to an extended timeframe in comparison to rats. This notion stems from comparing metabolic rate data and biochemical markers in the CNS between animals of different sizes (Kwon et al., 2010). Larger animals, such as pigs, might thus also be needed to model spinal injuries and treatment strategies (Kwon et al., 2015), including the development of improved surgical procedures for decompression and of novel methods for stem cell transplantation (Jones et al., 2012; Iwanami et al., 2005).

The fact that experimental spinal cord injury in rodents is such a robust and reliable model that it also allows the assessment of quite modest degrees of treatment-induced functional improvement has been argued to perhaps be another disadvantage (Reier et al., 2012). Injuries in humans, and the recovery from these injuries, are so heterogeneous that smaller improvements of function might be difficult to detect, unless very large trials are carried out (Wu et al., 2015).

The robustness of the rat spinal cord injury model has allowed extensive analysis of the pathology of spinal cord injury. However, our knowledge of human spinal cord injury pathology is more fragmented. As mentioned above, the period of BSCB permeability is well defined for rats after a spinal cord injury, but in humans with a spinal cord injury it is not. Interestingly, a recent study assessed blood–brain-barrier permeability by imaging contrast agents in humans after traumatic brain injury and revealed increased permeability for 5 days (Jungner et al., 2015). Another example is the deposition of ECM proteins, which in rats can be both a hindrance and a promoter of spontaneous repair; however, its composition remains largely unknown in humans (Buss et al., 2007). The field would benefit from a better understanding of the pathology of spinal cord injury in humans, in order to assess where similarities in pathology exist between the different animal models and to determine how pathological events differ over time.

Insult to the spinal cord initiates a multitude of cellular processes that develop over time. Hence, a combination of treatments is likely to be needed to make spinal cord injury a treatable disorder. Studies in rats have shown that both sequential combination and combining interventions at the same time might improve functional recovery. Combining neuronal stem cells with ten different growth factors (Lu et al., 2012) is a promising example of such a combinatory approach. Systems-level studies also offer an alternative, genomic and proteomic view, and provide us with a better understanding of spinal cord injury at the molecular level (Anderson et al., 2016; Didangelos et al., 2016). However, few attempts have been made to date to understand the course of pathology that follows spinal cord injury, or the effects of treatment on this pathology, using tools such as RNAseq and advanced proteomics. Systems-level studies might also provide further insight into the different consequences of spinal injuries between particular species.

## Conclusion

Experimental spinal cord injury studies in rats have allowed researchers to tackle the many aspects of pathology caused by the injury. However, much remains to be understood concerning how different aspects of rat pathology (and the pathology of other animal models in spinal cord injury research) relates to human pathology. Although the rat model has its limitations, few other models of neurological disorders and diseases are translationally as relevant and robust as those based on rats, not least with respect to functional parameters. The availability of additional genetically modified rats might strengthen its usefulness further. Many therapeutic interventions have progressed towards becoming candidate treatments for spinal cord injury, based on rat studies (Table 1), and some of these are currently being assessed in clinical trials.
